# Integrated functional visualization of eukaryotic genomes

**DOI:** 10.1186/1471-2105-7-348

**Published:** 2006-07-18

**Authors:** Rohit Ghai, Hannes Lindemann, Trinad Chakraborty

**Affiliations:** 1 Institute of Medical Microbiology, Faculty of Medicine, Justus-Liebig University, Frankfurter Strasse 107, 35392 – Giessen, Germany

## Abstract

**Background:**

Increasing amounts of data from large scale whole genome analysis efforts demands convenient tools for manipulation, visualization and investigation. Whole genome plots offer an intuitive window to the analysis. We describe two applications that enable users to easily plot and explore whole genome data from their own or other researchers' experiments.

**Results:**

STRIPE and GFFtool (General Feature Format Tool) are softwares designed to support integration, visualization and exploration of whole genome data from eukaryotic genomes. STRIPE, in addition to providing a highly customizable and interactive data plot, provides access to numerous well-selected databases with updated information on all genes of a genome. GFFtool provides a user-friendly solution to integrating experimental data with the genomic information available in public databases. They also obviate the need for users to maintain large annotation resources, as they link to well-known resources using standard gene and protein identifiers.

**Conclusion:**

The programs provide the user with broad genomic overviews of data distribution, fast access to data of interest, and the ability to navigate speedily from one resource to another, and gain a better understanding of result of whole genome analysis experiments.

## Background

The continuously growing availability of genomic information exercises pressure on the systems used to capture it and on users concerned with its interpretation. Analysis of large scale genomic data is a demanding task, requiring extensive input from diverse sources of biological significance, statistical methodologies and data exchange standards. To answer interesting biological questions, biologists need accessible interfaces that enable convenient visualization of information, searching multiple databases and flexible maneuvering within the data. When confronted with the lists of significantly differentially expressed genes from the microarray experiments performed, it is important to get a feel for the genome-wide distribution of the data and to be able to quickly navigate between diverse sources of information. Visualization on a genomic scale is also helpful in identifying and representing clusters of genes that are co-regulated and map close to each other in the genome. There are several examples of regions in the genome where genes implicated in the same biological processes are clustered together on the genome, e.g the cytokine-receptor cluster on mouse chromosome 16 [1], and a group of cytokine related genes associated with IL-4 on mouse chromosome 11 [2]. Chromatin remodeling events control transcription of closely mapped genes, and chromosomal clustering may point to regions where such events are actively induced.

**Figure 1:**
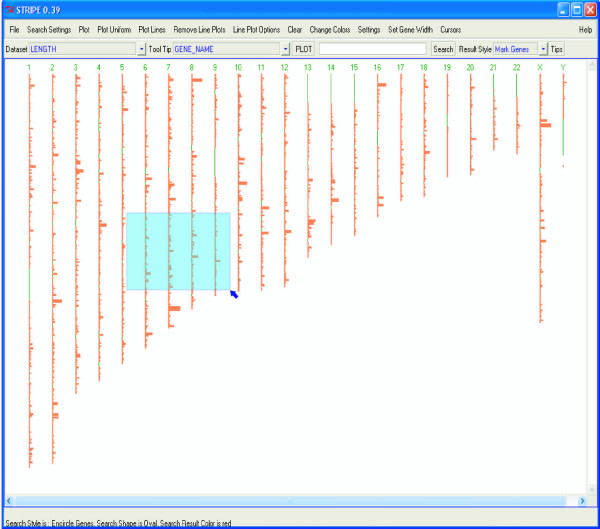
The figure shows all the genes in the human genome and their length plotted with STRIPE. Similar plots can be created immediately for the mouse and the rat genome by selecting a pre-prepared file. The blue colored box represents a selected region of interest. The data plotted in the figure is the gene lengths of all genes in the human genome. Longer genes appear thicker than shorter genes, as the lengths are scaled to size both along the vertical chromosome and the horizontal axis.

Since existing visualization solutions were not sufficient for our needs, we designed and implemented two programs, STRIPE and GFFtool for integrating, visualizing and exploring whole genome data. We feel that the solutions developed here will interest a broad range of scientists in different laboratory settings working with large scale genomic data sets from microarray, proteomic or even computational methods. We have made both tools freely available to academic users on our website. Currently, the human, mouse and rat genomes are available for immediate use. Figure 1 shows a plot of the entire human genome in STRIPE. The chromosome numbers and designations are indicated at the top of each chromosome. The gene lengths of all genes are plotted. Longer genes are thicker than shorter genes as the length of the genes here is scaled along both the vertical and the horizontal axes. The blue rectangle is a selection rectangle. It is possible to zoom in to a selected region by simply selecting a portion of the plot.

Although a few tools that have the ability to provide such plotting capability exist currently, none of them are sufficiently user-friendly or provide ways of extracting additional biological information about genes of interest. Users have to depend on extensive bioinformatics capabilities in order to get to the point of plotting the data. However, even after the data is plotted only limited interaction with the data is possible. Caryoscope[3] provides a genome-wide view of microarray data and some linking capabilities to the web. However, in spite of the provided guidelines, some prior experience of handling data from varied databases and with scripting languages such as Perl or Python is required. Similarly although it is possible to export appropriately formatted files from the Stanford Microarray Database[4], this is of little help to researchers who are interested in visualizing data from their own experiments, that may or may not be from microarrays. Caryoscope offers the advantage of being fully scriptable and easy to embed in a workflow, but has the disadvantage of being less useful as a platform for data exploration. Other applications, such as SeeGH [5], and CGHanalyzer [6], are designed for viewing dual channel array data derived from comparative genomic hybridization studies. ChromoViz [7] is implemented as an R package for visualizing genomic data. Although it is possible to plot several datasets for each chromosome at a time, obtain a karyotype plot for the chromosome in question, and explore data by zooming, the search capability is limited and web linking to publicly available databases is absent.

## Implementation

STRIPE and GFFtool have been programmed in Perl/Tk. We have tested both successfully on Windows, Linux and Solaris operating systems, but caution that some online resources may work better on the Windows platform. We recommend a minimum of 512 MB RAM to run the programs.

## Results and discussion

We describe the features of both programs, and provide illustrative examples of data plotting and exploration.

### GFFtool features

It is important to be able to integrate experimental data with annotation sources to be able to ask relevant biological questions. We use the well defined GFF (General Feature Format, [8]) file format for representing data. The program GFFtool was designed to collate our microarray data together with the annotations and locations of the genes while STRIPE provides an interactive environment for exploring the data on the genomic landscape.

The GFF format is well suited to store both numerical and textual data for genes and their features. Choosing a format is not enough though, and one must be able to easily create, modify, query and check if the data is in the correct format as well. GFFtool provides a number of methods to deal with the GFF file. Importantly, firstly it allows users to create their own GFF file for the genome of their choice. Detailed instructions for creating a new GFF file for a genome are provided in the program manual. For the large number of researchers interested in human, mouse and rat genomes, pre-prepared GFF files have already been created and may be used for browsing the genomes even without integrating it with any other data. All GFF files distributed with the programs have been created using GFFtool itself, using data distributed by NCBI and EBI. These files contain ~27,000 genes for human, ~26,000 genes for mouse and ~22,000 genes for the rat genomes.

In addition, integrating more data to an existing GFF file is a simple, one-step procedure. As both programs use standard NCBI Gene Database identifiers, once the experimental data has been linked to these identifiers, the data can be immediately added to an existing GFF file to create a new GFF file containing the experimental data. The experimental data need only be in the commonly used tab-delimited form to be accepted by GFFtool. Moreover, GFFtool also provides methods to re-format a few popular data files distributed by NCBI and EBI (e.g. xrefs files from EBI, gene2pubmed files from NCBI).

GFFtool also allows one to query the GFF file and extract subsets of interest (e.g. query for all transcription factors in the human genome), and create a GFF file exclusively for this subset of genes. It is also possible to subset a whole genome GFF file based on a text file containing a gene list of standard NCBI Gene Database identifiers (e.g. differentially expressed genes in a microarray experiment). Obtaining NCBI Gene IDs for the genes is a pre-requisite for combining the user's data with the GFF files used. Such conversions may be routinely performed by several already available tools, e.g DAVID [9] and MatchMiner[10].

The data from a GFF file can also be exported to a tab-delimited or a comma-delimited format for use in other applications (e.g. like spreadsheets). Also, GFFtool includes a format checking feature which allows one to thoroughly check the file before plotting in STRIPE.

### STRIPE features

STRIPE allows easy searching, highlighting and importantly, brisk navigation through several diverse annotation sources for any gene. It uses the standard NCBI Gene Database identifiers as minimal units for plotting. The pre-prepared GFF files for the human, mouse and the rat genomes are ready to plot directly with STRIPE. These files contain several annotation fields, e.g. the NCBI Entrez Gene ID, Gene Name, Gene Length, Gene Symbol, Cytogenetic location, GenBank accession, and Swissprot ID for all genes that have been mapped to a definite chromosomal location. These fields are used to link the application to a variety of different publicly available databases. Additional annotation fields may also be added to a GFF file using GFFtool.

STRIPE offers up-to forty different methods for plotting and coloring the data. It is possible to plot raw, zero-centered, mean-centered, log-transformed, and mean log-transformed data. One can plot a histogram plot and then overlay on it several line plots. Each plot can be colored in three different ways: using any user-defined color, a defined COLOR column in the GFF file, or a separate tag file that allows different groups of genes (e.g. genes belonging to the same biological process category as defined by Gene Ontology, or genes in the same pathway) to be appropriately labeled and colored. Upregulated and downregulated genes may also be colored differently at any time. In the absence of any user data, one can create and navigate a plot based only on the locations of the genes. This plot can also be colored as described above. It is useful when the user is mainly interested in knowing the location of the genes and exploring the genome.

**Figure 2:**
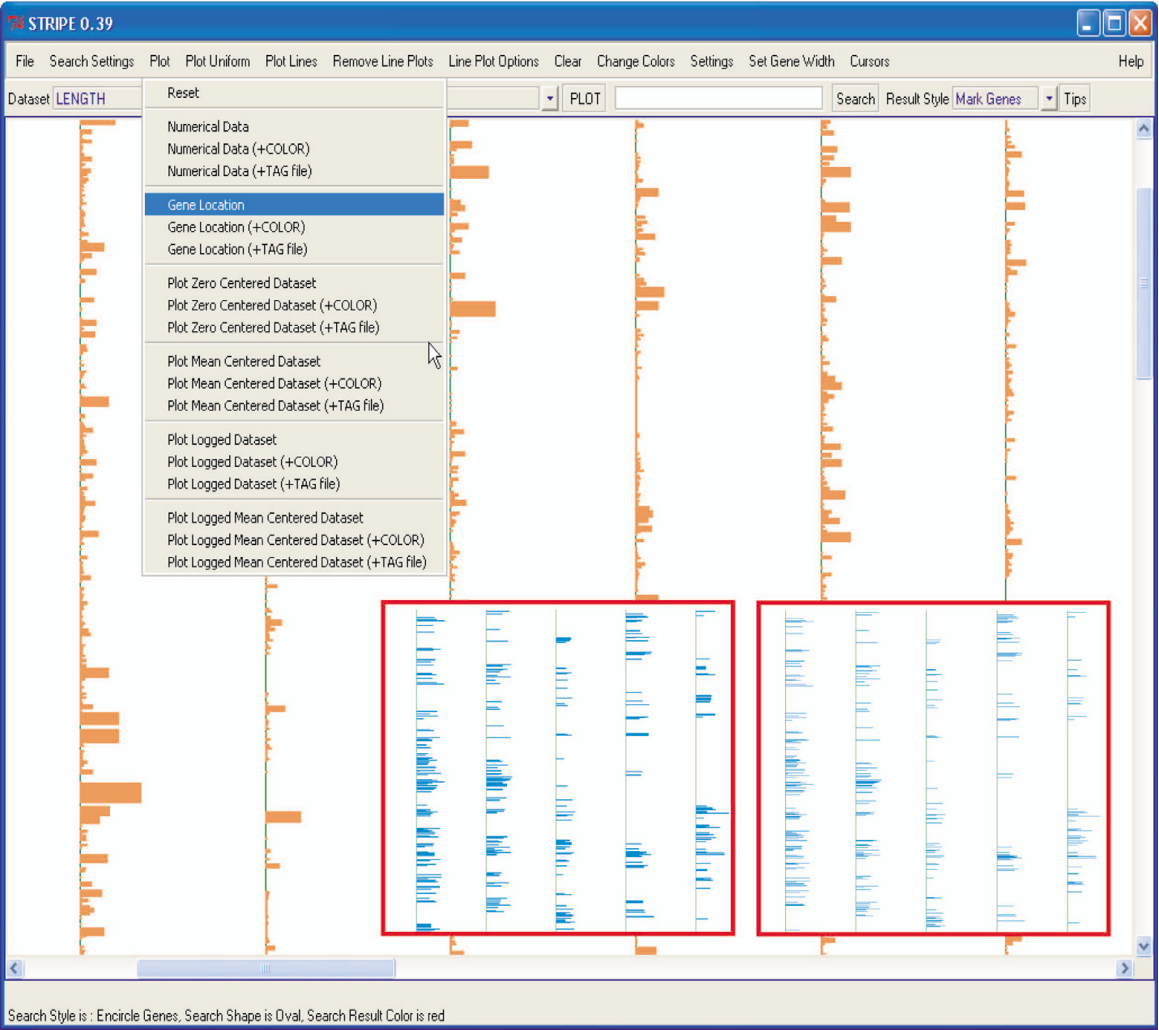
A zoomed in view of a few chromosomes is shown here. The plot menu shows the different choices for plotting a bar-chart in STRIPE. The two insets show a comparison between plotting data with (left inset) and without gene lengths (right inset) respectively, scaled along the chromosome. It is possible to plot the genes on the chromosomes in two ways, one in which the gene length is accurately scaled along the chromosome (giving the appearance of thick lines, which may be misunderstood for clusters of genes), and another in which the gene lengths are ignored and a line is drawn at the exact center of the gene. The latter gives a better view and helps avoid the misunderstanding that there are clusters. This is helpful in visual identification of possible gene clusters.

It is possible to choose a data column from the GFF file, values from which are displayed whenever one places the mouse cursor on any gene as a tool tip. In addition, the cytogenetic band and the value in the plotted data set are always displayed. Pressing and dragging the right side mouse button over a group of genes activates the zooming and lassoing feature. The selected area is zoomed in immediately for a closer examination. Figure 2 shows this in detail for a part of the plot. It is easy to see the bar-chart like plots in this view. The various options available for plotting are also shown in the plot pull-down menu. This menu is used when one desires to also scale the gene length along the chromosome. STRIPE provides two ways of creating each plot. One is to plot a data value associated with a gene as a horizontal rectangle on a vertical chromosome, where the horizontal axis of the rectangle corresponds to the data value and the vertical axis to the length of the gene. This is shown in the zoomed in view in Figure 2 where the gene lengths are scaled along the chromosome. The menu shown in the plot is used when one desires to have an idea of the actual gene lengths as well. The other method is to use a line perpendicular to the vertical chromosome. The Plot Uniform menu is used when one needs to ignore the gene length. In this case, this line is drawn at the exact midpoint of the gene and the gene length is disregarded. We have noticed that excluding the gene length during visualization provides a more reliable view of potential clusters of genes as the width of each gene is constant. An illustrative comparison is shown in the two insets in Figure 2. In the left inset the gene lengths have been plotted and in the right inset a single line is drawn at the center of each gene. A large number of spurious clusters can thus be visually filtered. It must be noted that this method does not override any statistical method used to identify clusters, but rather, acts as an aid to visualizing and exploring potential clusters. We suggest that any statistical tests for overrepresentation on the data be performed prior to its incorporation into STRIPE.

**Figure 3:**
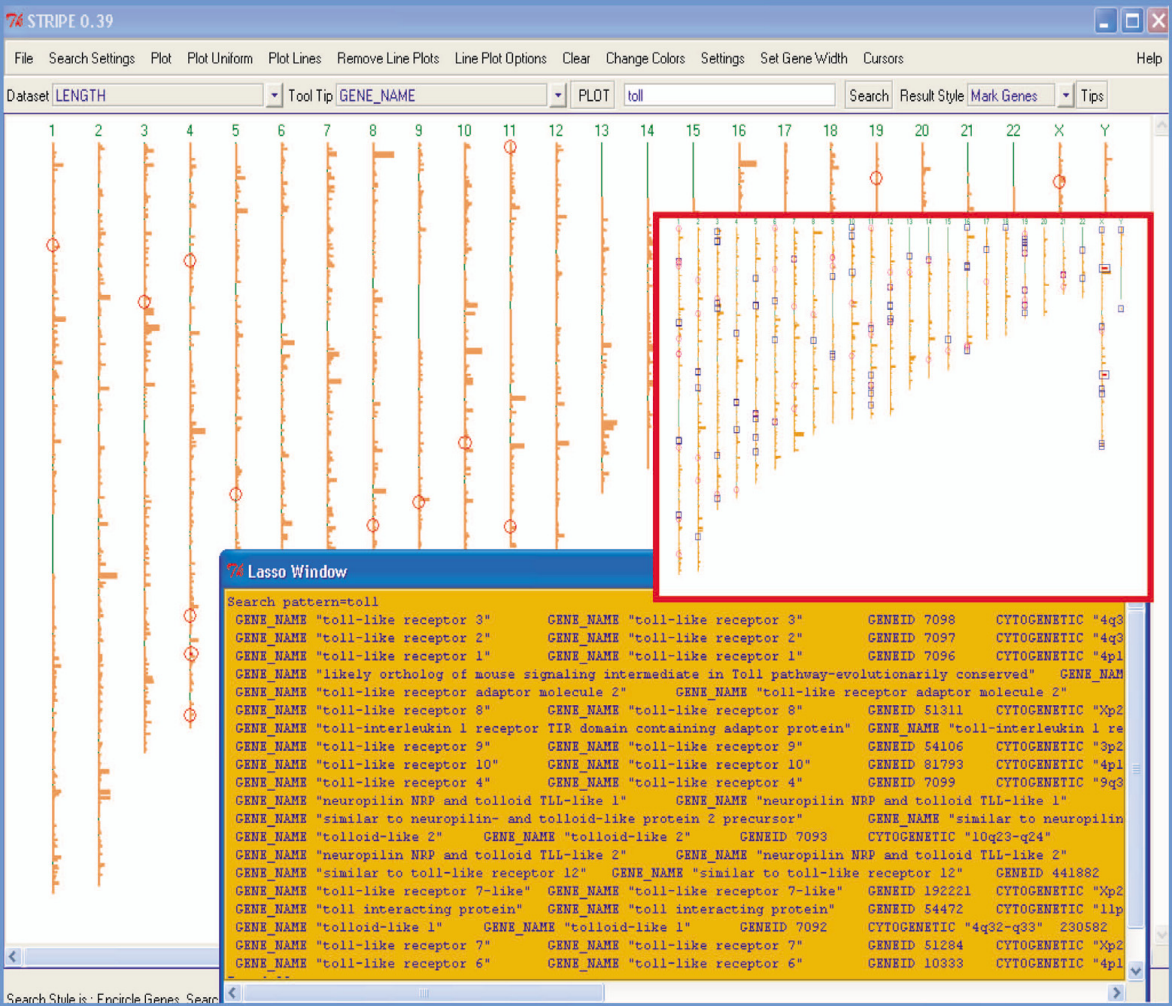
Search functionality in STRIPE. It is possible to perform a free-text or a regular expression based search on the entire genome and identify genes of interest. It is also possible to perform as many searches on a plot and mark up different search results in different colors and shapes. Shown in the figure are search results for the keyword "toll" in the human genome. Shown in the inset is how two different searches may be highlighted in the same plot (blue rectangle: keyword interleukin, red oval: keyword interferon).

Intuitive PERL-based regular expression searches may be carried out in the displayed tool tip column (e.g. searching the GENE_NAME column for genes matching the text "transcription factor") and the search results can be highlighted immediately on the plot. Highlighting can be done in many different ways (encircling the gene by an oval, circle or a rectangle, or changing its color). The colors of highlighting search results can be customized before each search is performed. This provides the user with the ease of locating the genes of interest. Also, as any number of searches may be performed on a plot, different subsets of genes may be highlighted simultaneously on the plot. Figure 3 shows an example of the search functionality in STRIPE. In this example, a simple search for the word "toll" highlights all the gene names in the human genome that contain the word "toll". The toll-like receptors are pathogen recognition receptors and important components of innate immune defense. The matching genes are colored red on the plot and encircled by an oval for immediate identification. The lasso window is also shown with details of the all the search results obtained with the pattern. The inset also shows the results of how two different search results may be highlighted in the plot. The blue rectangles are a search result for the word "interleukin" and the red ovals are the search results for the word "interferon". These search results can be saved to a text file. Plots may be saved as postscript files that can be read easily using standard graphics programs.

It is easy to reset the plot to its original state, and then select another area for zooming in. The lasso window provides detailed information on genes each time a search is performed or the plot is zoomed in. "Panning" on the canvas is done by pressing and dragging the left mouse button.

**Figure 4:**
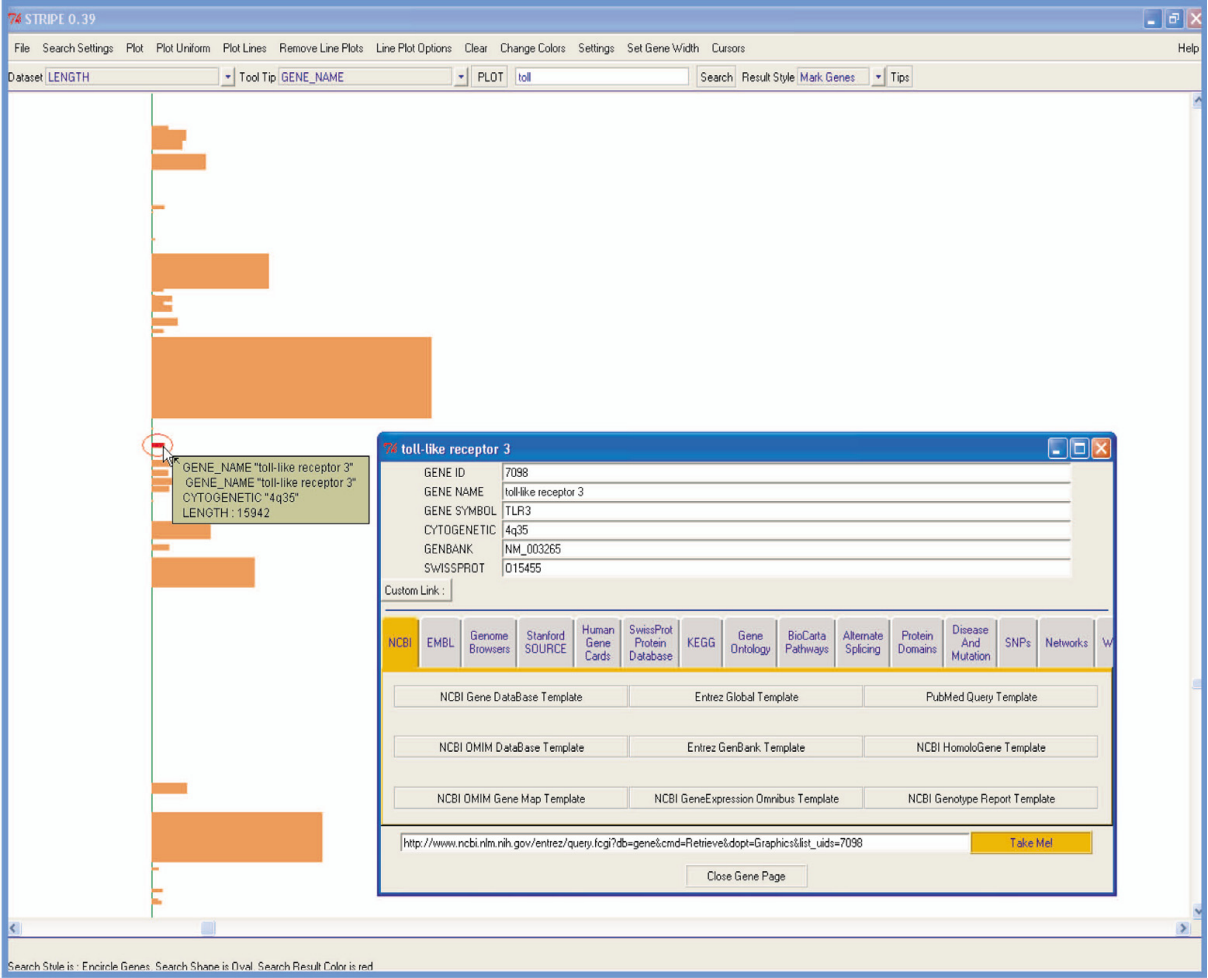
A zoomed in view of a search result for the keyword "toll" is shown here. Search results are shown encircled by a oval, and are colored in red. Both the color of the matching results, and how the result is encircled (oval, circle or rectangle) can be customized before searching, allowing several searches to be performed on the same plot. Also shown is the gene page window which provides well-organized access to the several information resources for each gene.

An important feature of STRIPE is the linking of the experimental data to a variety of biological databases. Using the database identifiers specified in the GFF file, STRIPE links to databases providing information on the gene, nucleotide and protein sequence information, single nucleotide polymorphisms, homologous genes across species, available PubMed literature, pathway information, gene expression data in public databases, genotype reports, chromosome maps, gene ontology categories, alternate splicing data, protein domains information, neighboring gene network associations, and genetic association databases. A customized link to a website using a data column from the GFF file can be defined. Figure 4 shows a close up view of the toll-like receptor 3 gene in the human genome. The gene is colored red and encircled by an oval. The mouse over feature provides a small box that indicates the gene name, the cytogenetic location, and the data value at that point. A double click on any gene brings up a small gene page window which shows further annotation details on the gene, in this case, toll-like receptor 3. The NCBI GeneID, official gene name, official gene symbol, cytogenetic location, Genbank accession number and the Swissprot identifier, is shown in the upper part of this window. The lower part of the gene page provides access to over 50 resources on this gene. All resources have been classified for easier access. It is also possible to provide an identifier in the GFF file and use it to create a customized deep link to any user-defined resource.

STRIPE provides access to a large number of different resources for each gene. Basic information, e.g. name, gene symbol, cytogenetic location etc, is provided right away. Deep links to several NCBI databases are provided, e.g. PubMed, GenBank, OMIM, Gene Expression Omnibus (GEO), HomoloGene. Three choices for genome browsers are provided, the NCBI Map Viewer, UCSC Genome Browser, and the Ensembl Genome Browser, so that users interested in a different display can easily switch to any of these and access additional information available there. All of the important databases for protein domains, PFAM, Interpro and SMART are available. Gene Ontology searches are available from the GO database. Transcripts with known alternative splice variants can be looked up the in Alternative Splicing Database. Pathway information can queried from KEGG and BioCarta, while literature connections can be explored using PubGene.

**Figure 5:**
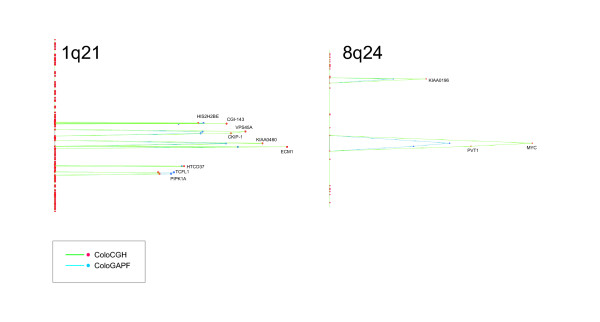
Plotting experimental data from study on a genome-wide analysis of palindrome prevalence using STRIPE. Two sets of data deriving from GAPF- and CGH-based arrays are plotted as line plots, one over the other. GAPF and CGH-based profiles of two selected regions (1q21 and 8q24) are shown in the figure. The Colo-CGH dataset is shown in green lines and with the genes as red, while the Colo-GAPF dataset is plotted with blue lines and blue spots as genes. The numerical values of the data are scaled along the horizontal axis.

In order to illustrate the utility of these programs we used data derived from a microarray-based genome-wide analysis of palindrome formation or GAPF [11]. The method described is a novel technique to detect the prevalence of palindrome-containing regions in cancer cells and to identify those regions harboring structural chromosome aberrations. The GAPF method was compared to conventional array comparative genome hybridization (CGH) in its ability to identify palindromes. The study concluded that DNA palindromes were frequent in human cancer and also provided evidence of possible clustering of palindromic sequences at specific chromosomal loci. In addition, GAPF was demonstrated to be more sensitive than array CGH in detecting DNA palindromes. Two datasets from the original publication, Colo_CGH and Colo_GAPF have been plotted together in line plots in STRIPE and are depicted in Figure 5. It is clearly seen that there is clustering of some of the data which is not immediately evident from tabular data as given in the supplementary section of the publication. The two insets show zoomed in regions of the plot. The left inset shows the chromosomal region at 1q21 where clustering of genes can be seen. The right inset shows a close up of the "MYC" gene at locus 8q24 encircled in the two line plots (both of which are also highlighted in the original publication). Once such a plot is created, it is publication-ready, fully interactive, zoomable and searchable and provides a starting point for navigation through several databases for each gene. This demonstrates how, in addition to providing tables, one may also easily provide overall visualization of the genomic distribution of the data.

**Figure 6:**
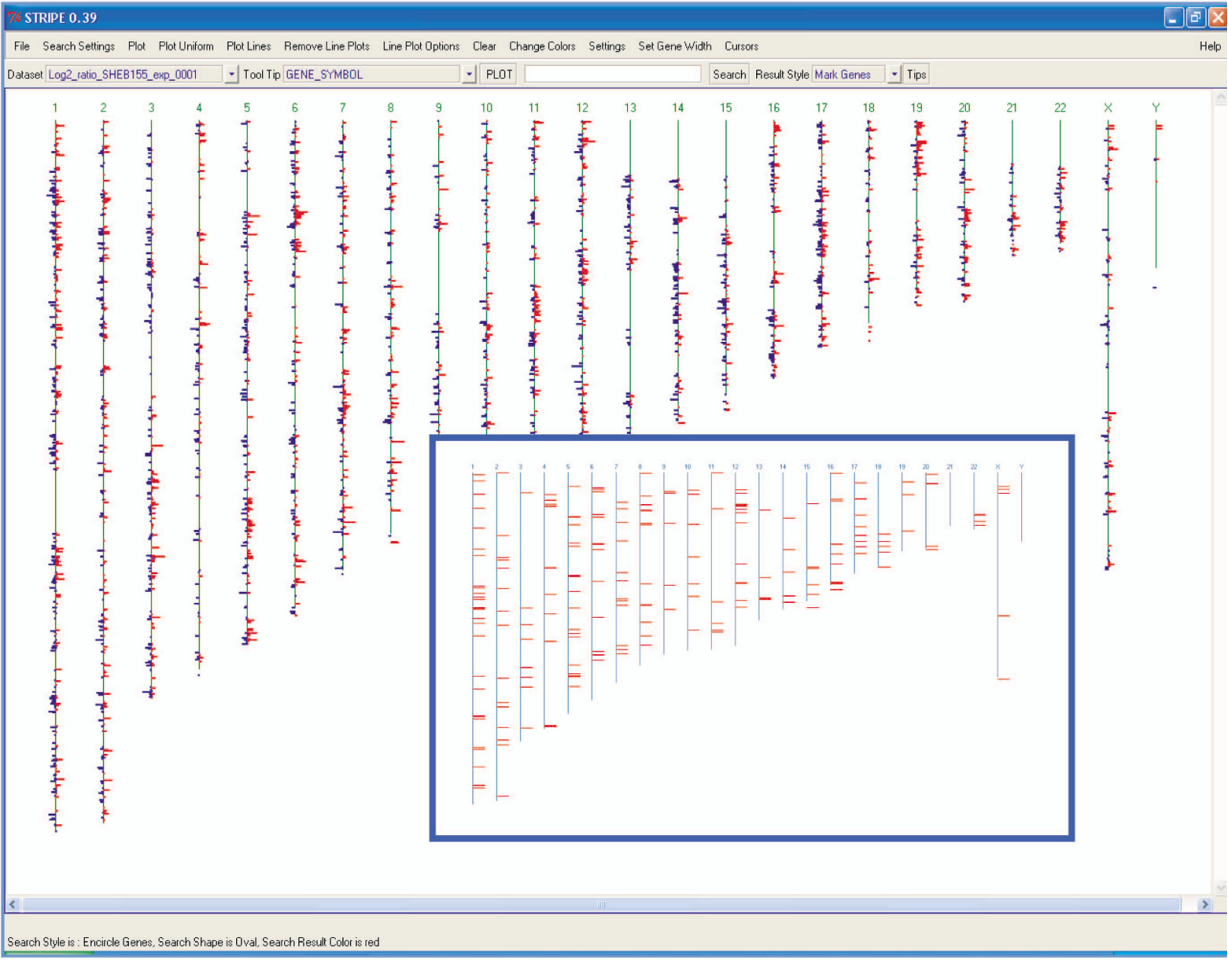
Plotting experimental data from a gene expression profiling study on renal cell carcinoma plotted with STRIPE. Upregulated genes are colored red and down regulated genes shown in blue. The inset depicts a genome-based representation of the genes identified as predictors for survival in conventional renal cell carcinoma. The numerical values of the data are scaled along the horizontal axis.

To provide another example of the program's capability to plot actual microarray data, we have taken data from a recent gene expression profiling study on cancerous renal cell carcinoma [12] and plotted it with STRIPE (see Figure 6). The data was retrieved from the Stanford Microarray Database and added to the human genome GFF file using GFFtool. A single experiment is shown here (SHEB155 exp001) and the log2 ratios have been plotted centered around zero. Upregulated genes are colored red and are plotted on the right sides of each chromosome while the downregulated genes are colored blue and are plotted on the left sides. It is also useful to plot identified differentially expressed genes separately purely as a means for reducing complexity and exploring hypotheses. The inset in the figure shows the cancer survival signature gene set identified by the authors plotted separately. As the plot is linked to the various resources for each gene, it provides an excellent gateway to explore each gene individually. The GFF files containing both the experiment and the cancer survival gene signature data are available with the programs.

## Conclusion

STRIPE and GFFtool are programs that make whole genome plotting easy for users, provide organized access to information resources, reducing time for manual navigation, and allow using a single plot as a gateway for exploration. Both STRIPE and GFFtool are still under development. Detailed documentation for both programs is available from the project home page.

## Availability and requirements

• **Project Name: **STRIPE and GFFtool

• **Project Home Page: **

• **Operating system(s): **Windows, Linux, Solaris

• **Programming Language: **Perl/Tk

• **Other requirements: **none

• **License: **Free for academic use

• **Any restrictions on use by non-academics: **Contact corresponding author for a license

## Abbreviations

**GFF: **General Feature Format

**NCBI: **National Centre for Biotechnology Information

**EBI: **European Bioinformatics Institute

**PFAM**: Protein Families Database

**KEGG**: Kyoto Encyclopedia of Genes and Genomes

**GAPF: **Genome wide analysis of palindrome formation

## Authors' contributions

RG conceived the program, wrote and tested it, prepared the manuals and the website. HL assisted in coding and testing both programs. TC oversaw the entire development process. RG and TC prepared the manuscript. All authors read and approved of the final manuscript.
